# Application of Computational Biology to Decode Brain Transcriptomes

**DOI:** 10.1016/j.gpb.2019.03.003

**Published:** 2019-10-23

**Authors:** Jie Li, Guang-Zhong Wang

**Affiliations:** 1 CAS Key Laboratory of Computational Biology, CAS-MPG Partner Institute for Computational Biology, Shanghai Institute of Nutrition and Health, Shanghai Institutes for Biological Sciences, Chinese Academy of Sciences, Shanghai 200031, China; 2 University of Chinese Academy of Sciences, Beijing 100049, China

**Keywords:** Brain transcriptome atlas, Computational analysis, Spatiotemporal pattern, Coexpression analysis, Single-cell analysis

## Abstract

The rapid development of high-throughput sequencing technologies has generated massive valuable **brain transcriptome atlases**, providing great opportunities for systematically investigating gene expression characteristics across various brain regions throughout a series of developmental stages. Recent studies have revealed that the transcriptional architecture is the key to interpreting the molecular mechanisms of brain complexity. However, our knowledge of brain transcriptional characteristics remains very limited. With the immense efforts to generate high-quality brain transcriptome atlases, new computational approaches to analyze these high-dimensional multivariate data are greatly needed. In this review, we summarize some public resources for brain transcriptome atlases and discuss the general computational pipelines that are commonly used in this field, which would aid in making new discoveries in brain development and disorders.

## Introduction

The mammalian brain is an evolutionary miracle that contains well-organized molecules, cell types, and neuronal circuits in each subregion; some of these features are closely connected at both the structural and functional levels. Moreover, brain development is an intricate, highly regulated process that continues throughout embryonic growth, and these lifespan program codes are conserved among species [1]. The complicated properties of the brain are mainly reflected in the complexity of its transcriptomic architecture, including highly ordered gene expression and elaborate transcriptional regulation. For example, the majority of genes (>80%) are expressed in the mammalian brain [2], and the expression profiles of these genes show great variability during development, with the most remarkable changes occurring during development in prenatal and postnatal stages [3], [7]. On the other hand, brain tissues exhibit the smallest transcriptomic changes compared with other organs [8], [9]. Therefore, understanding the spatiotemporal characteristics of gene expression can offer valuable insights into brain functional specialization and the roles of key genes during brain development. Furthermore, analyzing the transcriptomic architecture of normal brain development and function is of vital importance to determine the causes of a variety of complicated neurological disorders.

In the last decade, many quantitative methods have been applied to explore the expression of individual genes, particularly the spatial and temporal patterns across the brain. The development of microarray analysis and various high-throughput sequencing technologies has made it possible to investigate the expression of genes in a high-throughput manner, yielding large datasets_._ Specifically, single-cell sequencing can be used to quantify the transcriptome of a single cell, providing major opportunities to parse the complex cellular composition of the brain. However, analysis of such high-dimensional data remains substantially complex and requires more effective and sophisticated computational methods and models. Recent progress in computational and systems biology fields has facilitated transcriptomic studies with high precision to obtain new insights into the transcriptional characteristics of the brain.

**Figure 1:**
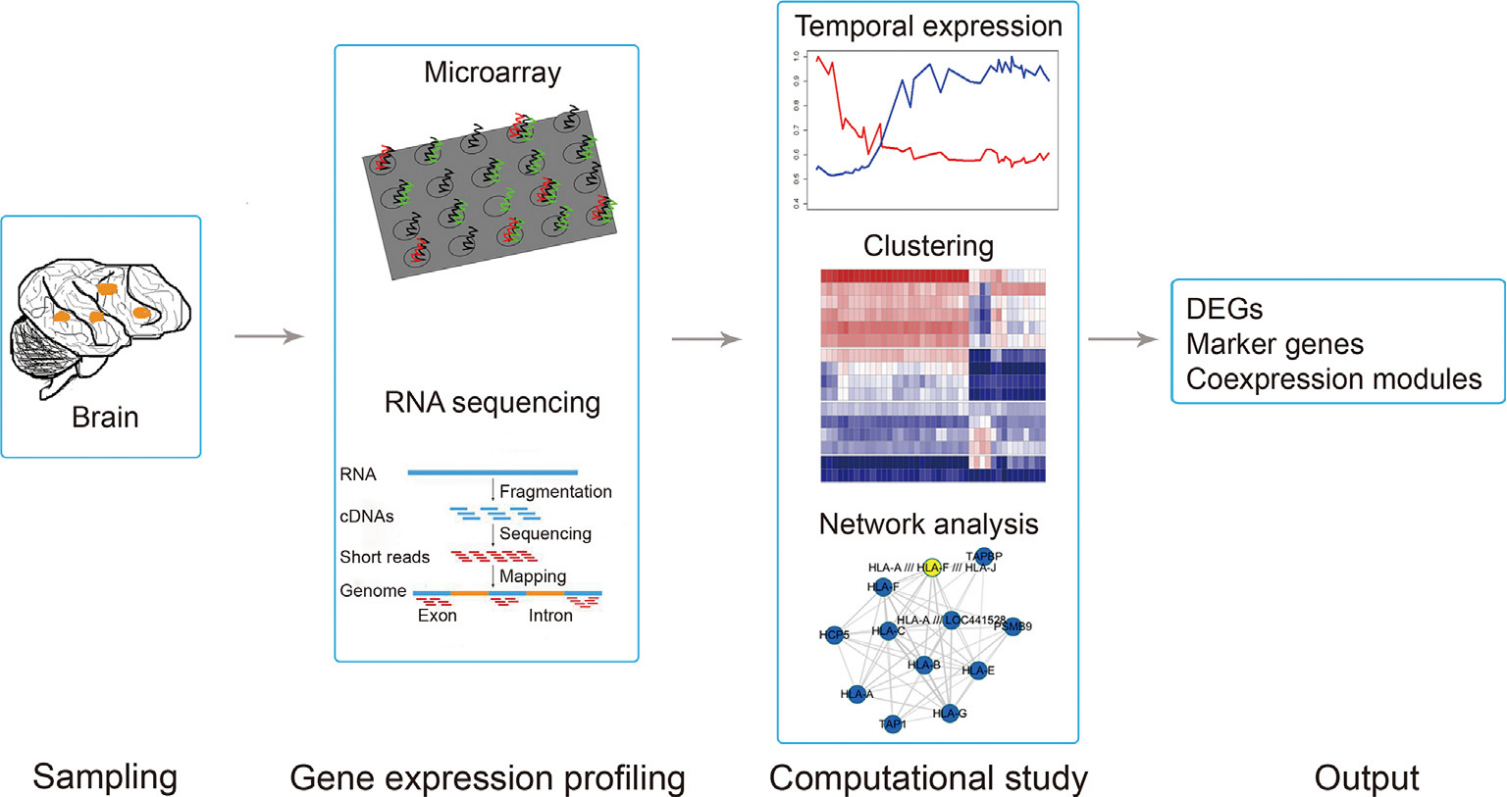
**General pipeline of computational analysis of the brain transcriptome** Brain samples are collected and the expression of all genes in each region is profiled by either microarray or next-generation sequencing. Then computational strategies are applied in order to identify DEGs, marker genes, or network co-expression modules. DEG, differentially-expressed gene.

In this review, we introduce a variety of brain transcriptome atlases and discuss how to apply computational methods to elucidate the relationships between gene expression and brain function as well as the relationships between brain development and disease. Many of these relationships have been discovered by following the general pipeline of brain transcriptome analysis (Figure 1). Finally, we state some limitations in recent transcriptome studies and offer some directions for future studies.

## Brain transcriptomic atlas resources

In the past decade, an increasing number of researchers have realized the importance of large-scale brain transcriptome data, and various countries have launched big brain research projects, which have greatly promoted the study of the molecular mechanisms of brain organization and function. The rapid development of high-throughput technologies has made it possible to quantify the expression of thousands of genes simultaneously. Currently, various brain transcriptome datasets from humans and other species can be obtained from different molecular platforms, such as microarray, RNA sequencing, and *in situ* hybridization (ISH). For rodents, the Gene Expression Nervous System Atlas (GENSAT) [10], [11] and GenePaint [12] have provided expression signals for thousands of genes in developing and adult mouse brains. However, compared with mouse brain atlases, the available human brain expression atlas is less abundant because there are more difficulties in obtaining, storing, and analyzing human postmortem brain tissues [13]. Fortunately, several studies have investigated gene expression variations among different brain regions [14], [15] and at different development time points [16], [3], [4], [5], [6], [7]. Furthermore, a series of transcriptome atlases of the developing and adult mouse brains [2], [17], the developing and adult human brains [18], [19], and the nonhuman primate (NHP) brains [20], [21], [22] have been released. Specifically, the Allen Institute for Brain Science (http://brain-map.org/) possesses comprehensive transcriptomic sources from mouse and human brains and is a great resource for many neuroscience fields [23]. To facilitate the application of these data, we have summarized some available brain transcriptome resources in Table 1. Notably, Jason et al. has provided a detailed user guide for some brain transcriptome databases in another review [24]. In this review, we include a series of data released recently. We believe that these available transcriptome data are essential components for investigating the complex molecular architecture of the brain at a large scale.

**Table 1:** Summary of major brain transcriptome resources

**Species**	**Sample**	**Age**	**Region**	**Method**	**Web link**	**Data access**	**Annotation**	**Refs.**
Mouse	Bulk tissues	Multiple	CNS	BAC transgenic; ISH	http://www.gensat.org/	URL	Spatiotemporal	[10], [11]

Mouse	Bulk tissues	Multiple	Multiple	ISH	http://www.genepaint.org	URL	Spatiotemporal	[12]

Mouse	Bulk tissues	Multiple	Multiple	ISH	http://www.emouseatlas.org/emage/	URL	Spatiotemporal	[25]

Mouse	Bulk tissues	Lifespan	Multiple	ISH	http://developingmouse.brain-map.org/	URL	Spatiotemporal	[17]

Mouse	Bulk tissues	Postnatal	NCX	MiD; RNA-seq	http://hbatlas.org/mouseNCXtranscriptome	SRP031888	Spatiotemporal	[26]

Mouse	Bulk tissues	Adult	NCX layers	RNA-seq	http://genserv.anat.ox.ac.uk/layers	GSE27243	Spatial	[27]

Mouse	Bulk tissues	Adult	Multiple	ISH	http://mouse.brain-map.org/	URL	Spatial	[2]

Mouse	Bulk tissues	Postnatal	Forebrain	FACS; PAN; microarray	www.ncbi.nlm.nih.gov/geo	GSE9566	Cell-type specific	[28]

Mouse	Bulk tissues	Adult	NCX	PAN; FACS; RNA-seq	http://web.stanford.edu/group/barres_lab/brain_rnaseq.html	GSE52564	Cell-type specific	[29]

Mouse	Bulk tissues	Adult	CNS	TRAP; microarray	http://genetics.wustl.edu/jdlab/csea-tool-2	GSE13379	Cell-type specific	[30]

Mouse	Bulk tissues	Embryonic	NCX	FACS; RNA-seq	http://decon.rc.fas.harvard.edu/	GSE63482	Cell-type specific	[31]

Mouse	Bulk tissues	Adult	HIP	Genetic labeling; RNA-seq	http://hipposeq.janelia.org	GSE74985	Cell-type specific	[32]

Mouse	Bulk tissues	Adult	Multiple	Genetic labeling; RNA-seq	http://neuroseq.janelia.org	GSE79238	Cell-type specific	[33]

Mouse	Single-cell	Postnatal	Brain; SC	SPLiT-seq	www.ncbi.nlm.nih.gov/geo	GSE110823	Spatiotemporal	[34]

Mouse	Single-cell	Adolescence	NS	FACS; 10X Genomics	http://mousebrain.org	SRP135960	Spatial	[35]

Mouse	Single-cell	Juvenile; adult	Multiple	scRNA-seq	http://linnarssonlab.org/oligodendrocytes/	GSE75330	Spatiotemporal	[36]

Mouse	Single-cell	Adult	NCX; HIP CA1	Fluidigm C1	http://linnarssonlab.org/cortex	GSE60361	Spatial	[37]

Mouse	Single-cell	Adult	HPA	Drop-seq	www.ncbi.nlm.nih.gov/geo	GSE87544	Spatiotemporal	[38]

Mouse	Single-cell	Adult	RB neurons	FACS; Drop-seq	https://portals.broadinstitute.org/single_cell	GSE81905	Spatial	[39]

Mouse	Single-cell	Adult	HIP	Div-seq (nuclei)	https://portals.broadinstitute.org/single_cell	GSE84371	Spatial	[40]

Mouse	Single-cell	Adult	V1 (NCX)	FACS; SMARTer	http://casestudies.brain-map.org/celltax	GSE71585	Spatial	[41]

Mouse	Single-cell	Adult	HIP	SMART-seq	www.ncbi.nlm.nih.gov/geo	GSE71485	Spatiotemporal	[42]

Mouse	Single-cell	Adult	STR	Mic-scRNA-seq; FACS-scRNA-seq	www.ncbi.nlm.nih.gov/geo	GSE82187	Spatial	[43]

Mouse	Single-cell	Adult	Multiple	Drop-seq	http://dropviz.org/	GSE116470	Spatial	[44]

Mouse	Single-cell	Adult	NCX	FACS; SMART-seq	www.ncbi.nlm.nih.gov/geo	GSE115746	Spatial	[45]

Mouse	Single-cell	Adult	HPA	MERFISH; Drop-seq	www.ncbi.nlm.nih.gov/geo	GSE113576	Spatial	[46]

Mouse	Single-cell	1–3 M; 21–22 M	Brain	10X Genomics	http://shiny.baderlab.org/AgingMouseBrain/	GSE129788	Temporal	[47]

Mouse	Mixed	Multiple	Multiple	Microwell-seq	https://figshare.com/s/865e694ad06d5857db4b	GSE108097	Spatiotemporal	[48]

Mouse	Mixed	Adult	Multiple	FACS; Microfluidic	https://tabula-muris.ds.czbiohub.org/	GSE109774	Spatial	[49]

Rhesus macaque	Bulk tissues	Lifespan	Multiple	LMD; microarray	http://www.blueprintnhpatlas.org/	URL	Spatiotemporal	[21]

Rhesus macaque	Mixed	Lifespan	Multiple	RNA-seq; 10X Genomics	http://www.evolution.psychencode.org/	PRJNA448973	Spatiotemporal	[50]

Human	Bulk tissues	Lifespan	Multiple	MaD; exon-array	http://hbatlas.org/	GSE25219; GSE13344	Spatiotemporal	[3], [5]

Human	Bulk tissues	Lifespan	Multiple	LMD; microarray; ISH; RNA-seq	http://www.brainspan.org/	URL	Spatiotemporal	[19]

Human	Bulk tissue	Lifespan	Multiple	Multi-omics	http://development.psychencode.org/	phs000755.	Spatiotemporal	[51]

Human	Bulk tissues	Adult	Multiple	MaD; LMD; microarray; ISH	http://human.brain-map.org/	URL	Spatial	[18]

Human	Bulk tissues	Lifespan	PFC	Microarray	http://braincloud.jhmi.edu	GSE30272	Temporal	[6]

Human	Bulk tissues	Fetal; juvenile; adult	NCX; HIP	PAN; RNA-seq	http://www.brainrnaseq.org/	GSE73721	Cell-type specific	[52]

Human	Single-cell	Fetal	PFC	SMART-seq2	www.ncbi.nlm.nih.gov/geo	GSE104276	Temporal	[53]

Human	Single-cell	Fetal	NCX	STRT-seq	www.ncbi.nlm.nih.gov/geo	GSE103723	Spatial	[54]

Human	Single-cell	Fetal	NCX	Fluidigm C1	https://cells.ucsc.edu/?ds=cortex-dev	phs000989.v3	Spatiotemporal	[55]

Human	Single-cell	Fetal; adult	NCX	Fluidigm C1	www.ncbi.nlm.nih.gov/geo	GSE67835	Spatiotemporal	[56]

Human	Single-cell	Fetal; adult	Multiple	Fluidigm C1	http://www.psychencode.org/	URL	Spatiotemporal	[51]

Human	Single-cell	Adult	NCX	Fluidigm C1(nuclei)	http://www.scap-t.org/	phs000833.v3.p1	Spatial	[57]

Human	Single-cell	Adult	Multiple.	snDrop-seq	www.ncbi.nlm.nih.gov/geo	GSE97942	Spatial	[58]

Human	Single-cell	Adult	MTG	SMART-seq v4	http://celltypes.brain-map.org/	URL	Spatial	[59]

Human	Mixed	Adult	Multiple	RNA-seq	https://www.gtexportal.org	URL	Spatiotemporal	[14]

Drosophila	Single-cell	Adult	Brain	10x Genomics; Drop-seq	http://scope.aertslab.org	GSE107451	Spatiotemporal	[60]

Drosophila	Single-cell	Adult	Midbrain	Drop-seq	https://www.ncbi.nlm.nih.gov/sra/SRP128516	SRP128516	Spatial	[61]

Zebrafish	Single-cell	Juvenile	Brain	GESTALT; Drop-seq	http://krishna.gs.washington.edu/content/members/aaron/fate_map/harvard_temp_trees/	GSE105010	Spatiotemporal	[62]

Zebrafish	Single-cell	Adult	Hab	FACS; 10X Genomics; SMART-seq2	http://stackjoint.com/zbrain/	GSE105115	Spatial	[63]

Multiple	Bulk tissue	Adult	Multiple	RNA-seq	http://www.psychencode.org/	PRJNA236446	Species	[64]

Multiple	Mixed	Multiple	Multiple	Multi-omics	https://www.encodeproject.org	URL	Integrative	[65]

*Note*: Web links for supporting resources are provided when available. Multiple means that samples were obtained from multiple species, tissues, brain regions, or at multiple time points. NCX, neocortex; HPA, hypothalamus; PFC, prefrontal cortex; HIP, hippocampus; Hab, habenular; RB, retinal bipolar; STR, striatum; CNS, central nervous system; SC, spinal cord; BAC, bacterial artificial chromosome; V1, primary visual cortex; MTG, middle temporal gyrus; MiD, microdissection; MaD, macrodissection; FACS, fluorescence-activated cell sorting; LMD, laser microdissection; PAN, immunopanning; TRAP, translating ribosome affinity purification; GESTALT, genome editing of synthetic target arrays for lineage tracing; ISH, *in situ* hybridization; MERFISH, multiplexed error robust fluorescence *in situ* hybridization.

## Analyzing brain-wide gene expression patterns

### Spatial and temporal gene expression analyses

**Figure 2:**
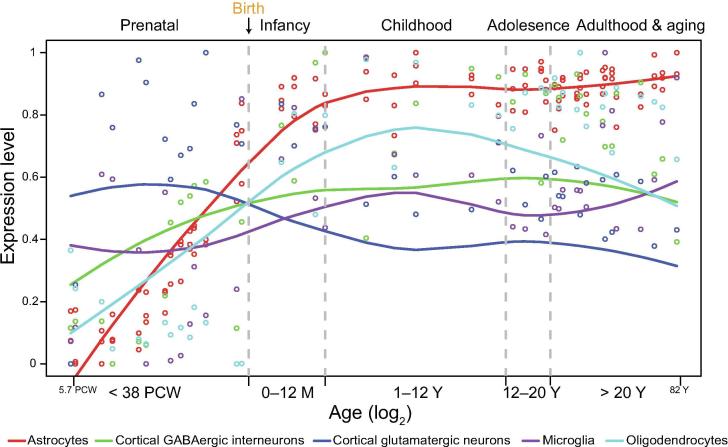
**Timeline of major human brain cell types based on gene expression trajectories** The X axis shows the developing time and the Y axis represents the relative gene expression level (percentage of maximum). The occurrence and progression of each cell type are reflected by the expression trajectories of the associated genes (data are from the Human Brain Transcriptome project [5]). Based on these trajectories, the prenatal and early infant stage is the most dynamic phase of different cell types. PCW, postconceptional weeks; M, months; Y, years.

One important aspect of brain complexity is that the brain is organized into multiple functional regions with distinct transcriptomic architectures. Therefore, a good strategy for studying the functions of a specific gene is to analyze its expression across different developmental stages and/or brain regions. Many transcriptomic analyses of prenatal and postnatal tissues have shown that the intricate principles of human brain development can be revealed by carefully surveying spatial and temporal gene expression [3], [4], [5], [6], [7]. For example, Kang et al. used a high-throughput exon array to characterize the spatial and temporal transcriptomes of the human brain [5]. The authors collected more than 1000 postmortem brain samples, covering 16 different regions of the human brain (the hippocampus, striatum, cerebellar cortex, amygdala, mediodorsal nucleus of the thalamus, and 11 neocortical areas). These tissue samples spanned 15 periods from the prenatal stage (5.7 weeks after conception) to the aging stage (82 years old), making this collection one of the most comprehensive collections of brain transcriptome data. This work provides new insights into the spatiotemporally regulated patterns of brain-related genes and their co-expression relationships [5]. The data also show that the predominately regulated stage is the prenatal stage (70.9% genes are spatially differentially expressed, 89.9% genes are temporally differentially expressed, and 70.0% of all expressed genes are regulated in both patterns) [5]. Furthermore, based on the spatial and temporal transcriptome data, researchers can obtain the developmental trajectories of key genes, such as marker genes of different cell types (Figure 2). For brain development and neurodevelopmental disorders, an important problem that needs to be solved is when and where the key genes are expressed and how such expression is disrupted in neurodevelopmental disorders. These gene expression trajectories are valuable resources to dissect the molecular mechanisms underlying the functional specialization of brain regions. More importantly, these trajectories can also contribute to understanding the causes of various neurodevelopmental diseases.

In addition to analyzing spatiotemporal expressional patterns, some groups have considered temporal gene expression dynamics among different brain regions, reflecting the functional specialization of brain regions. Using the mouse brain, Liscovitch and Chechik [66] identified differentially expressed genes in multiple brain regions and determined how regional dissimilarities changed over time. In this study, they calculated the dissimilarity for each pair of regions, defined as 1 – Pearson’s correlation coefficient. Their results suggest an hourglass pattern in which dissimilarities increase greatly in early prenatal development, decrease to a minimum at birth, and increase again after birth [66]. Notably, they observed a significant postnatal specialization in the mouse cerebellum, and a similar phenomenon was also observed in human brains [66]. In another study related to the human cortex, a temporal hourglass pattern consisting of three major phases was discovered by Pletikos and the colleagues [7]. Prenatal development is the first phase and is characterized by the highest number of differentially expressed genes. The pre-adolescent phase is the second phase, showing less divergent regional gene expression and a more synchronized gene expression pattern. The last phase is adolescence, showing increased regional differences again [7]. This cup-shaped transcriptional divergence pattern is repeatedly observed in the transcriptome of developmental brains from both humans and macaques. Interestingly, the transcriptional divergence between human and macaque brains also exhibits a cup-shaped pattern, as reported in two recent studies [50], [51]. These temporal differences in gene expression among different brain regions provide valuable insights into the specialization of brain function.

Unlike the aforementioned studies, Colantuoni et al. focused on only one region, the dorsolateral prefrontal cortex (PFC, BA46/9), a newly evolved area that is involved in executive functions such as working memory, emotion, cognition, decision-making, and social behavior [67], [68], [69], [70]. In this study, 269 human brain samples spanning gestational week 14 to aging (>80 years old) were analyzed [6]. Interestingly, approximately three quarters of genes showed reversed expression between the prenatal stage and early postnatal stage, and these reversals were also observed between the prenatal stage and much later in life (approximately 50 years old) [6].

Because tissue samples from the human brain are invaluable, and most existing studies cannot cover all important brain areas and developmental time points, NHPs, such as chimpanzees and rhesus monkeys, are preferred over mice for parsing the development and functions of the human brain. A comprehensive transcriptome atlas of the developing brain of rhesus monkeys was proposed by Bakken and colleagues [21]. This atlas includes anatomical reference data (with magnetic resonance imaging [MRI]), ISH gene expression data (cellular level), and developing transcriptome data (covering 10 stages throughout the lifespan). Using this highly precise transcriptional map, Bakken et al. found that dramatic changes in gene expression occurred in both progenitor cells and neurons in the prenatal stage [21]. Furthermore, by comparing the gene expression conversion between humans, rhesus monkeys, and rats, they confirmed that rhesus monkeys share more similar gene expression with humans than with rats (22% versus 9% of genes showed different expression trajectories in rats and humans versus rhesus monkeys and humans, respectively) [21], indicating that NHPs are valuable for investigating human-specific changes in brain development.

In addition to characterizing gene expression changes in different regions and tracing expression trajectories of important genes during brain development in a specific species, comparative transcriptomic analysis can also provide valuable insights into brain evolution. A set of studies have compared brain gene expression between humans and other species to capture conserved features and human-specific changes. For example, by constructing and comparing the co-expression networks of the brain between humans and mice, Miller et al. found that the network properties are conserved between humans and mice [71], which is consistent with the results of previous studies [72]. Furthermore, the human-specific modules identified are correlated with Alzheimer’s disease. For NHPs, Xiao et al. compared region-specific gene co-expression networks between humans and macaques to investigate brain functional divergence [73]. By calculating the topological features of these networks, a structural difference was found; human genes are more closely connected to form functional modules [73]. Similarly, Sousa et al. compared the transcriptome profiles of humans, chimpanzees, and rhesus macaques (247 samples from 16 regions) and found that regions from the same species are clustered together based on miRNA and mRNA expression, except for the cerebellum [64]. These results also showed that differentially expressed genes with human-specific patterns, including transcription factors and neurotransmitter biosynthesis enzymes and receptors, play important roles in neural circuit formation [64].

### Brain-wide coexpression modularity analysis

In the aforementioned study, Kang et al. found that the brain transcriptome tends to organize into co-expression networks that are implicated in distinct biological processes [5]. Generally, genes that share similar expression patterns among samples or time points are defined as co-expressed genes, and there is a high possibility that these genes are involved in similar biological processes [74]. Thus, identifying the co-expressed network based on expression similarity is a powerful method to obtain context-specific functional annotations.

In practice, the key fundamental part of co-expression analysis is how to measure gene expression similarities. Generally, people choose similarity measures according to the purpose of their studies, such as Pearson’s correlations, Spearman’s correlations, partial correlations, mutual information, Euclidian distances, Cosine similarities, and probabilistic measures. The most widely used are correlation-based measurements. For example, NeuroBlast can identify genes with similar three-dimensional spatial expression based on Pearson’s correlations [75], and the Spearman’s correlation coefficient can be used to analyze co-expression gene pairs in the mouse brain [76]. Another example is a recent study that analyzed the co-expression pattern of chromodomain helicase DNA-binding protein 8 (*CHD8*), a key autism-associated gene [77]. This study showed that *CHD8* is widely expressed in both cortical and subcortical structures, although its expression density decreases during development in both human and macaque brains. Moreover, significant enrichment of autism genes was observed in CHD8-correlated genes [77].

Generally, unsupervised clustering and network analyses are appropriate for exploring molecular interactions between a set of genes that may have similar biological functions or be involved in similar pathways. As an unsupervised method, hierarchical clustering is widely used to group genes and samples. Gofflot et al. applied unsupervised hierarchical clustering to explore the expression of nuclear receptors (NRs) in 104 brain regions [78]. They found that anatomical brain structures are organized in three main clusters in favor of the existing taxonomy models of brain, and NRs are clustered in two major groups, with distinct expression patterns [78]. Besides clustering, another approach is constructing a co-expression network in which the nodes are co-expressed genes and the edges represent co-expression relationships of gene pairs with or without weights. The most widely used co-expression network in practice is weighted gene co-expression network analysis (WGCNA), a computational approach to identify network modules based on the topological profiles of a co-expression network [79]. In WGCNA, there is an eigengene for each module, which represents the overall expression of that module, and hub genes can be identified further based on the connectivity of the module members. In this way, the module’s function can be inferred based on the function or enrichment analysis of those hub genes [79]. In neuroscience, this method has been widely applied to construct transcription networks of the mammalian brain. For example, Oldham et al. used WGCNA to compare the network conservation between human and chimpanzee brains [80]. They observed that functional modules of the cerebral cortex are less likely to be conserved during evolution than those of other brain regions [80]. Moreover, other studies applied WGCNA to identify modules associated with distinct cell types and functions or corresponding to distinct brain regions in the developing and adult brains of mice, rhesus monkeys, and humans [5], [17], [18], [81]. For example, Hawrylycz et al. identified 13 co-expression modules with specific anatomical distributions to characterize the transcriptional variations across the adult human brain [18].

Complex neurological disorders are not caused by a single gene but multiple dysregulated genes, which may converge in the same dysregulated biological processes. With the increasing number of samples taken into consideration, genome-wide association studies have linked an increasing number of variants with complex neurological and neuropsychiatric disorders, including autism spectrum disorders (ASDs) [82], [83], [84], [85], [86], [87], schizophrenia [88], [89], and Alzheimer’s disease [90], [91]. In this context, analyzing co-expressed genes with known disease-related genes can provide an avenue to dissect the molecular underpinnings of complex neurological disorders. Ben-David and Shifman used WGCNA to analyze the co-expression relationships of rare and common autism variants and found two modules affected by rare and common variations corresponding to the plasticity of synapses and neurons and the areas of learning and memory, respectively [92]. In another study, Menashe et al. used cosine similarity as a measurement of expression similarity and constructed a co-expression network of autism genes in the mouse brain [93]. These studies demonstrated that autism-related genes are preferentially co-expressed. Moreover, Menashe et al. identified two modules in which autism-related genes are highly connected and overexpressed in a specific brain region, the cerebellar cortex [93]. These abovementioned studies have shown a link between the network of autism-related genes and specific brain regions. Furthermore, researchers can use co-expression analysis to examine when and where specific genes are expressed and how they change during specific biological processes, such as neuron differentiation and maturation, which may provide another view for research into neurodevelopmental disorders. Some studies have been conducted in this field. For example, Parikshak et al. constructed brain developmental-related WGCNA networks based on the BrainSpan dataset (www.brainspan.org) and mapped ASD-related and intellectual disability-related genes onto different modules [94]. Their results demonstrated that modules significantly enriched in ASD genes are involved in distinct biological functions, such as the regulation of synaptic development [94]. They further found that ASD genes are preferentially located in superficial cortical layers and expressed in glutamatergic projection neurons [94]. In another study, Mahfouz et al. analyzed 455 autism genes to identify their shared pathways [95]. They showed that modules containing large numbers of ASD genes are related to biological processes involving synaptogenesis, apoptosis, and GABAergic neurons [95]. All of these studies demonstrated that the co-expression network is a powerful strategy to reveal the biological functions of disease-risk genes.

### Cell type-specific gene expression analysis

The brain is the most heterogeneous organ, in which diverse cell types are assembled into distinct but highly connected circuits and regions. Thus, it is possible to identify functional regions and neural cell types based on their transcriptional architecture, not on their morphological and electrophysiological properties. However, in general transcriptome studies, RNAs are extracted from tissue samples and examined *en masse*, which means the characteristics of a specific cell type are missing, further limiting the utility of bulk transcriptome data, since the expression changes that occur in rare cell types may not be detected. Therefore, it is necessary to directly quantify the transcriptome of a specific cell type. In practice, various methods, such as laser-capture microdissection, immunopanning, fluorescence-activated cell sorting, manual cell sorting, and transgenic engineering, are used to identify and isolate specific cell types. A detailed review has compared these methods [96], and another review has provided an overview of existing studies combining these methods and high-throughput transcriptomes to explore cell-specific expression patterns [24].

In addition, great efforts have been made to extract cell type-specific or region-specific patterns from bulk brain transcriptome data. For example, Kirsch et al. proposed a method to detect layer-specific gene expression in the mouse cerebellum [97]. In this work, the authors used a histogram of local binary patterns to represent each gene’s ISH image and predicted the localization based on a two-level classification. First, a classifier based on a support vector machine was trained to identify images of specific layers. Then, genes were classified based on multiple-instance learning [97]. Similarly, Li et al. developed another method (scale-invariant feature transform) to detect cell type-specific genes from ISH images [98]. Zeng et al. applied a deep convolutional neural network to the developing mouse brain [99]. In this work, they used two approaches to extract features from ISH data, *i.e.*, the invariant image feature descriptors method and regularized learning method [99]. All of these studies have demonstrated that computational approaches, particularly feature exacting methods, are helpful for detecting cell type-specific and/or region-specific genes. However, these methods are based on some known marker genes of specific regions, layers, or cell types, and the accuracy of the results needs to be improved. A better choice is characterizing the total transcriptome at the single-cell level and grouping cells into distinct populations based on their transcriptional pattern, as discussed below.

### Single-cell gene expression analysis

Combined with physical isolation of specific cell types and computational analysis of brain cell pools, the transcriptional atlas of specific cell types can be depicted. However, the accuracy needs to be improved, and heterogeneity still exists. Recently, advances in the isolation of single cells have made it possible to generate the transcriptome of a single cell, and a series of single-cell transcriptome data have been released (Table 1). Researchers can use single-cell RNA-seq (scRNA-seq) to discriminate distinct cell populations, identify new and rare cell types, and trace cell developmental trajectories.

The mammalian brain is viewed as the most complicated organ largely due to the heterogeneity of diverse specialized cell types. Since scRNA-seq can describe the transcriptome from a single cell and the same types of cells are likely to share similar expression patterns, researchers can assign individual cells to distinct cell populations based on the similarity of the transcriptome, not just based on the expression of marker genes. scRNA-seq has shown great power to explore the heterogeneity of cells in the brain (Table 2). In practice, unsupervised clustering methods, including hierarchical clustering, k-means clustering, principal component analysis, and t-distributed stochastic neighbor embedding, are widely used to identify cell subpopulations. Notably, it is better to apply these clustering methods to differentially expressed genes or highly variable genes. For example, Zeisel et al. measured the transcriptomes of 3005 cells from two regions of the adult mouse brain, that is the primary somatosensory cortex (S1) and hippocampal CA1 region [37]. First, they selected 5000 genes based on a series of strict criteria. Then, they used an algorithm called BackSPIN to cluster genes and cells simultaneously, and identified 47 subclasses of nine major clusters (S1 and CA1 pyramidal neurons, interneurons, oligodendrocytes, astrocytes, microglia, vascular endothelial cells, mural cells, and ependymal cells). Next, Zeisel and colleagues extracted specific markers of each cell population. Some of these markers are well known, while some are novel, such as *Gm11549* specific for S1 pyramidal cells, *Spink8* specific for hippocampal pyramidal cells, and *Pnoc* specific for interneurons [37]. Notably, the general analysis assumes that the cell types are abundant. If the cells are small in number or rare, it is a challenge to discriminate them from the cell populations. To solve this problem, Grun et al. proposed RaceID, which uses transcript counts to identify the rare and abundant cell types in complex cell pools [100]. Overall, RaceID has two major steps. First, k-means clustering is applied to the similarity matrix, and the cluster number is determined from the gap statistic [101]. Then, outlier cells are identified followed by rare cell type identification [100]. Using RaceID, Grun et al. identified a novel marker for enteroendocrine cells, *Reg4* [102].

**Table 2:** scRNA-seq studies revealing multiple cell types in the brain

Species	Region	No. of total cells	No. of neuronal cells				No. of non-neuronal cells				Ref.
			GABAergic	Glutamatergic	SN	NPC	Immune	Oligodendrocyte and OPC	Astrocyte	Vascular	
Zebrafish	Brain	66,000	47,822 (45)			7404 (9)	672 (2)	1064 (3)	0	1007 (4)	[62]
Mouse	S1; HIP CA1	3005	300 (16)	1351 (12)	0	0	90 (5)	811 (6)	210 (2)	270 (6)	[37]
Mouse	V1	1679	664 (22)	609 (19)	0	0	22 (1)	59 (2)	43 (1)	27 (2)	[41]
Mouse	STR	1208	0	0	904 (2)	7 (1)	119 (2)	56 (3)	107 (1)	82 (4)	[43]
Mouse	HPA	14,437	1392 (18)	906 (15)	0	0	891 (1)	5484 (4)	1148 (1)	1610 (3)	[38]
Mouse	V1; ALM	23,822	10,534 (61)	11,905 (56)	0	0	136 (2)	189 (5)	583 (1)	476 (4)	[45]
Mouse	HPA	31,299	15,042 (43)	3511 (23)	0	0	906 (3)	8857 (9)	856 (2)	1123 (6)	[46]
Mouse	Brain	50,212	3726 (10)	1037 (7)	0	248 (2)	4448 (8)	15,463 (8)	6931 (5)	3884 (8)	[47]
Mouse	Brain; SC	156,049	128,953 (54)				621 (2)	10,087 (7)	13,481 (4)	2907 (4)	[34]
Human	NCX	3127	905 (8)	1928 (8)	0	0	0	0	0	0	[57]
Human	PFC	2309	701 (8)	1057 (7)	0	290 (9)	68 (4)	107 (4)	71 (3)	0	[53]
Human	NCX	4213	968 (8)	1538 (4)	0	103 (2)	830 (7)	82 (2)	112 (2)	161 (2)	[54]
Human	VC; FC; CBL	35,289	7809 (13)	18,045 (14)	0	0	756 (1)	5727 (3)	2524 (2)	219 (1)	[58]
Human	MTG	15,603	4164 (45)	10,525 (24)	0	0	0	551 (2)	291 (2)	0	[59]

*Note*: The numbers in parentheses indicate the number of subpopulations. S1, primary somatosensory cortex; V1, primary visual cortex; STR, striatum; HPA, hypothalamus; ALM, anterior lateral motor cortex; SC, spinal cord; NCX, cerebral cortex; PFC, prefrontal cortex; VC, visual cortex; FC, frontal cortex; CBL, cerebellum; MTG, middle temporal gyrus; SN, spiny neuron; NPC, neuronal progenitor cell; OPC, oligodendrocyte progenitor cell; HIP CA1, cornu ammonis area1 of hippocampus.

Another important implication of scRNA-seq is tracking cell trajectories during a dynamic process, such as neuronal differentiation. However, it is difficult to determine which cell type at time point *n* progresses to a cell at time point *n* + 1 in scRNA-seq data since the cell is completely consumed. In addition, the cells collected from a sample may not be completely synchronized. Some algorithms have been developed to address these problems, and these algorithms can be generally divided into two classes. These include pseudotime ordering methods, such as diffusion pseudotime (DPT) [103], single-cell topological data analysis (scTDA) [104], Wanderlust [105], Waterfall [42], and Monocle 2 [106], and probabilistic branch models, such as single-cell clustering using bifurcation analysis (SCUBA) [107] and temporal assignment of single cells (TASIC) [108]. In practice, pseudotime ordering methods usually require dimension reduction first, followed by reconstruction of cell trajectories in the lower dimension space, in which graph analysis is usually required, including the minimum spanning trees and principal curves. Recently, Lin et al. proposed a method called continuous-state hidden Markov model (CSHMMs) to infer branching topology and assign cells to the correct branches [109]. In neuroscience, these aforementioned methods are widely used to track cell trajectories during brain development. For example, Zhong et al. performed monocle pseudotime analysis [110] of human prefrontal cortex development and revealed the following development branches for neural progenitor cells, including two paths to intermediate progenitor cells and one late path to outer radial glia (RG) [53]. In another study, Polioudakis et al. explored the diversity and lineage of cell types during human neocortex development. First, they identified 16 distinct cell populations from ∼40,000 cells and then performed pseudotime ordering analysis [111]. Moreover, they found ordered transitions during neural progenitor differentiation, such as RG transitioning to intermediate progenitors (IPs) and IP transitioning to newborn migrating neurons [111].

Although scRNA-seq has shown extraordinary superiority in characterizing neuronal cell types and their distributions, some issues should be considered; for example, high variability in levels of the detected transcripts. In the future, advanced methods are required to improve the coverage of the transcriptome and preserve the physiological microenvironment of cells.

### Integrative analysis of brain transcriptome and neuroimaging data

In recent years, neuroimaging technology has been greatly developed, providing an unprecedented opportunity to associate molecular variance with macroscopic changes in the brain. Although a large number of brain transcriptome atlases are available, most lack the capability to cover the entire brain, except the Allen Brain Atlas (ABA). ABA is an anatomically comprehensive atlas, comprising 3702 transcriptomes from six adult brains. Importantly, ABA contains MRI data and Montreal Neurological Institute coordinate data [18], allowing researchers to integrate the relationship between spatial variation at the molecular level and observed neuroimaging phenotypes. Recently, many studies have suggested that gene expression is related to the functional connectivity of the brain. In an early study in this field, Goel et al. explored whether there is a relationship between gene expression and anatomical brain regions [112]. They extracted structurally connected regions based on magnetic resonance (MR) diffusion tractography and found no direct relationship between structural connectivity and similar expression patterns at the individual level [112]. In another study, Wang et al. used fractional amplitude of low-frequency fluctuations, a region-specific index, to associate genes with a network called the brain functional activity default mode network, which contains brain regions that exhibit coherent functional magnetic resonance imaging (fMRI) signal fluctuations under the resting state [113]. They found that these related genes are preferentially expressed in neurons and the expression of these genes is downregulated in the brain of autistic patients [114]. In another similar study, Richiardi et al. found that functionally connected regions have similar gene expression patterns via mapping ABA expression data to 14 functional networks [115]. Furthermore, they identified 136 genes driving the relationship that are significantly enriched in ion channels [115]. In addition to investigating the relationship between variations in gene expression and variations in structural/functional connections of the brain, other researchers have shifted their focus to the relationship between structural changes in the brain and gene expression patterns. One example is a study by Whitaker et al., in which the authors explored the underlying mechanism of brain structure maturation during adolescence [116]. Specifically, they collected 297 samples and measured the thickness and myelination of the cortex via MRI. Their results demonstrate a significant association between the shrinkage and myelination of the cortex and the gene expression patterns of dorsoventral areas [116].

Notably, integrative analysis of transcriptome and imaging data often involves many variables, which requires sophisticated data processing. Over the years, various software and tools have been developed to perform such analyses [117], [118], [119], [120]. However, the accuracy and consistency of the results obtained are largely affected by the choice of these tools. Recently, a practical guide for key procedures in analyzing HABA data has been proposed to facilitate studies in this field [121]. In the future, developing methodological guidelines to for more accurate results is still necessary.

## Limitations and future directions

### The resolution of brain ISH data

Although great progress has been made in quantifying gene expression in the brain, several aspects in the field regarding the analysis of the spatial and temporal patterns of the brain must be improved. One key problem is the low resolution of human brain expression imaging data. Although cellular-level resolution is possible in the original ISH data (∼1 µm), much higher resolution data are desired for genome-wide data used in three-dimensional (3D) space (∼200 µm) [13]. The low resolution poses challenges to investigations into the detailed characteristics of the organization of the brain. Many researchers have attempted to develop new approaches to solve this problem. For example, Ramsden et al. realigned mouse ISH data using nonlinear regression model, which increased the resolution to approximately 10 µm [122]. Using this method, the expression levels of genes that can define the border and layers of medial entorhinal cortex were identified [122]. In the future, more general methods are needed to integrate spatial gene expression data into the standard 3D space.

### Expression of non-coding RNAs

Current transcriptome data of the brain mainly focus on the expression of protein-coding genes (mRNAs), whereas the expression features of non-coding RNAs (ncRNAs) are often ignored. In recent years, a series of studies have shown that ncRNAs are of great importance in brain development and neurological disorders [123], [124]. In an early study, Mercer et al. analyzed the ISH data from the adult mouse brain and identified a large number of ncRNAs (849 transcripts) [125]; most of these ncRNAs have specific expression profiles in different brain regions and cell types [125]. In another study, Fertuzinhos et al. focused on the transcriptional differences among neocortex layers and how these differences change during brain development. As a result, they profiled the temporal transcriptomes of the mouse S1 region, including protein-coding genes and ncRNAs [26]. Similarly, Ziats and Rennert explored the roles of microRNAs (miRNAs) during human brain development, and identified miRNAs with spatial- and/or sex-dependent expression and their putative targets [126]. Further functional analysis revealed that these differentially expressed miRNAs are involved in many basic developmental events and neurological disorders [126]. All the aforementioned studies demonstrate that necessity of exploring the expression of ncRNAs and their regulatory basis throughout brain development.

### Integrative analysis with other neuro-omics data

The rapid development of high-throughput sequencing technologies provides not only transcriptome atlases but also other omics atlases of the brain. Transcriptomes reflect the abundances of RNA, whereas epigenomics data, such as DNA methylation and histone modifications, describe the underlying regulatory mechanisms of gene expression. Additionally, proteomics data provide a more reliable readout of gene expression. With the available isolation of more homogeneous brain samples and great advances in single-cell analysis [127], [128], multiple omics data of the brain can be obtained. For example, Illingworth et al. explored the interindividual variability in the human brain methylome and found that compared to other brain regions, the cerebellum has a distinct methylation pattern, which is consistent with the results of transcriptome analysis [129]. In another study, Vermunt et al. identified *cis*-regulated elements across brain regions, and further analysis of coregulation of the enhancer network revealed hidden cell type and functional information [130]. Furthermore, the psychENCODE project aims to construct a neurobiological epigenetic landscape of adult and developing human brains that are normal or diseased [131]. Based on these high-dimensional multi omics, it is necessary to develop systematic approaches to conduct integrative analyses. Integrating different multi omics datasets can help us better explore the molecular mechanisms underlying complex phenotypes and neurological disorders.

## Conclusion

In recent years, the hypergrowth of next-generation technologies has enabled high-throughput transcriptome measurement of the brain throughout its main developmental stages. The accompanying brain transcriptome atlases are also valuable sources to reveal the molecular architecture of the brain. Computational methods are important to decode these high-dimensional transcriptome data. Combined with transcriptome data and appropriate approaches, the relationships among spatial and temporal gene expression, the complex brain traits, and neurological disorders can be studied. However, with the emergence of new data and the limitations of current data (such as low resolution and the lack of non-coding genes), developing new computational methods remains necessary to overcome limitations and identify new molecular underpinnings of the brain. Furthermore, new systematic approaches are needed to conduct integrative analyses of transcriptomic data and other neuro-omics data.

## Competing interests

The authors have declared no competing interests.
